# Effect of complex training on lower limb strength and running economy in adolescent distance runners

**DOI:** 10.3389/fphys.2025.1718150

**Published:** 2025-11-12

**Authors:** Shiping Yu, Shengqing Zhou, Daibin Peng, Dongli Jie

**Affiliations:** 1 College of Professional Tennis, Wuhan City Polytechnic, Wuhan, China; 2 School of Physical Education, Wuhan Sports University, Wuhan, China; 3 Hubei Leisure Sports Development Research Center, Wuhan, China; 4 College of Physical Education and Health, Guangxi Normal University, Guilin, China; 5 College of Liberal Education, Hubei Three Gorges Polytechnic, Yichang, China; 6 Wuhan Asia General Hospital, Wuhan, China

**Keywords:** contrast training, energy cost, muscle power, plyometric exercise, endurance performance

## Abstract

**Background:**

While traditional resistance training has been widely used in distance running training, the effects of complex training combining heavy resistance and plyometric exercises on adolescent runners remain unclear. Understanding the impact of complex training on running economy and lower limb strength could provide valuable insights for optimizing training programs for young athletes.

**Purpose:**

To examine the effects of an 8-week complex training program on lower limb strength and running economy in adolescent distance runners compared to traditional resistance training.

**Methods:**

Thirty-two male adolescent distance runners (age: 16.75 ± 0.68 years) were randomly assigned to either a complex training group (CT; n = 16) or a resistance training group (RT; n = 16). Both groups completed their respective training programs three times per week for 8 weeks, in addition to their regular endurance training. Running economy at three speeds (12, 14, and 16 km/h), maximal oxygen uptake (VO2max), blood lactate concentration, One-repetition maximum (1RM) squat strength, countermovement jump (CMJ), squat jump (SJ), drop jump (DJ), and reactive strength index (RSI) were assessed before and after the intervention.

**Results:**

The CT group showed significantly greater improvements in running economy at all speeds (p < 0.001) compared to the RT group. Both groups demonstrated significant improvements in 1RM squat strength (CT: p < 0.001; RT: p < 0.001), CMJ (CT: p < 0.001; RT: p < 0.001), and SJ (CT: p = 0.005; RT: p < 0.001). The CT group exhibited superior improvements in CMJ peak power (p = 0.010), DJ performance (p = 0.017), and RSI (p < 0.001). Blood lactate concentrations at submaximal speeds decreased significantly more in the CT group compared to the RT group (p < 0.05).

**Conclusion:**

Complex training appears to be more effective than traditional resistance training for improving both running economy and power-related performance measures in adolescent distance runners. The combination of heavy resistance and plyometric exercises may provide superior neuromuscular adaptations that enhance both strength and running efficiency.

## Introduction

Distance running performance in adolescents is fundamentally governed by two critical physiological parameters: lower limb strength (Blagrove et al., 2018) and running economy (Gäbler et al., 2018). While traditional training approaches have predominantly focused on aerobic conditioning, emerging evidence suggests that the integration of strength and power training could potentially optimize these key performance determinants (Gäbler et al., 2018).

Running economy, defined as the energy cost at a given submaximal speed, represents a crucial determinant of distance running performance (Saunders et al., 2004; Fletcher and MacIntosh, 2017). Research indicates that superior running economy can differentiate between athletes of similar maximal oxygen uptake (VO_2max_) levels, potentially providing a competitive edge in distance events (Barnes and Kilding, 2015; Shaw et al., 2014). This advantage arises because running economy reflects the integrated efficiency of multiple physiological systems—including biomechanical, neuromuscular, and metabolic components—that determine how effectively oxygen is converted into forward motion (Barnes and Kilding, 2015; Shaw et al., 2014). Athletes with superior running economy expend less energy and accumulate less metabolic fatigue at a given speed, enabling them to sustain higher intensities for longer durations (Fletcher and MacIntosh, 2017; Fletcher et al., 2010). Moreover, improved stiffness regulation and motor unit recruitment efficiency contribute to reduced ground contact time and enhanced stride mechanics, further optimizing energy utilization during running (Fletcher and MacIntosh, 2017). These neuromuscular factors are trainable qualities that can be effectively enhanced through strength and power training, which in turn may further improve running economy. Traditional endurance training approaches, while effective for cardiovascular adaptation, may not optimally address the neuromuscular components that influence running economy (Beattie et al., 2017; Denadai et al., 2017).

Lower limb strength plays a dual role in distance running performance (Stricker et al., 2020). First, it provides the fundamental force production capability necessary for propulsion during each stride. Second, it contributes to running economy by improving neuromuscular efficiency and reducing the relative intensity of each stride. However, conventional strength training methods have shown inconsistent results in adolescent populations, possibly due to insufficient integration with sport-specific movement patterns (Lloyd et al., 2014; Stricker et al., 2020).

Complex training represents a potentially superior approach by combining heavy resistance exercises with biomechanically similar plyometric movements (Maio Alves et al., 2010). This training methodology theoretically capitalizes on post-activation performance enhancement (PAPE) effects and may enhance both strength and power development more effectively than traditional resistance training alone (Cormier et al., 2022). However, some studies have examined the effects of traditional resistance and plyometric training in adolescent runners. For instance, Blagrove et al. (2018) demonstrated that plyometric training improved running economy and performance in adolescent endurance athletes, suggesting that such training modalities can be beneficial even in this population.

Previous research has demonstrated that carefully implemented strength training can benefit adolescent athletes without compromising their growth or increasing injury risk (Faigenbaum et al., 2016). However, the specific effects of complex training on running economy and lower limb strength in adolescent distance runners have not been thoroughly investigated. This knowledge gap is particularly important given the unique physiological characteristics and training responses of adolescent athletes (Lloyd et al., 2016).

Therefore, this study aims to examine the effects of an 8-week complex training program compared with traditional resistance training on lower limb strength and running economy in adolescent distance runners. We hypothesize that complex training will provide superior improvements in both parameters compared to traditional resistance training, potentially offering a more effective training strategy for this specific population.

## Materials and methods

### Participants

Sample size calculation using GPower software (version 3.1.9.7; Franz Faul, University of Kiel, Germany) determined that 24 participants were needed, based on the following parameters: α = 0.05, power (1-β) = 0.8, effect size f = 0.4, and statistical analysis using repeated measures ANOVA with within-between interaction. Accounting for a potential 20% dropout rate, 32 healthy male adolescent distance runners were recruited from teams in Guizhou Province, China.

Participants were post-pubertal male runners aged 16–18 years with a minimum of 2 years of distance running experience. Inclusion criteria were: (1) current membership on a provincial team; (2) minimum 3 years of systematic training; (3) healthy status enabling completion of all physical fitness tests; (4) no lower limb injuries in the previous 3 years; (5) regular competition experience at county, regional, national, or international level in middle-distance events (800–3000m); and (6) consistent training volume of ≥35 km per week during the 3 months preceding the study.

Prior to enrollment, participants and their parents were thoroughly briefed on the study purpose, design, potential risks, and possible discomfort. All provided written informed consent before the study commenced. None of the participants were taking medications that could affect physical performance. This study was reviewed and approved by the Research Ethics Board of Wuhan Sports University on 3 January 2025 (Approval number: 2025076). The participants were recruited between January 4 and 8 January 2025, and the intervention was conducted from January 9 to 9 April 2025. All procedures were in accordance with the Declaration of Helsinki.

### Procedures

**FIGURE 1:**
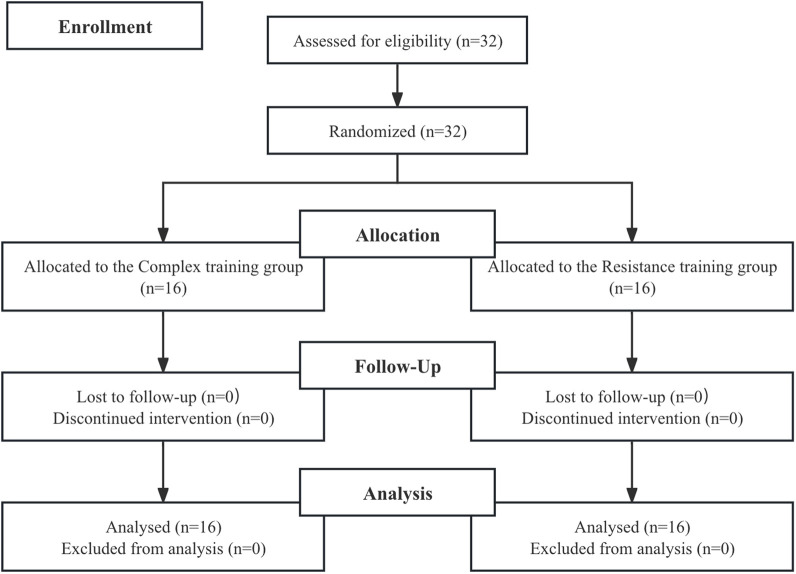
Flow chart of the progress through the phases of the study according to the CONSORT statements.

This study employed a between-group repeated-measures design to investigate the effects of two distinct resistance training modalities on lower limb strength and running economy in adolescent distance runners. The study spanned 12 weeks in four phases: 2 weeks of preparatory strength training, 1 week of pre-testing, 8 weeks of resistance training intervention, and 1 week of post-testing. Initial assessments included body composition, One-repetition maximum (1RM) back squat, squat jump (SJ), countermovement jump (CMJ), drop jump (DJ), running economy (RE), and VO_2max_. Participants were then randomly assigned to either a complex training group (CT; n = 16) or a traditional resistance training group (RT; n = 16), using a simple randomization procedure. A computer-generated random number sequence was created by an independent researcher, and allocation was concealed in sealed opaque envelopes until group assignment. Baseline physical characteristics of both groups are presented in Table 1 and Figure 1.

**TABLE 1:** Physical characteristics of participants in the CT and RT control groups.

Group	Age (year)	Height (cm)	Mass (kg)	Body fat (%)	BMI (kg·m^2^)
RT (n = 16)	16.75 ± 0.68	177.8 ± 3.8	62.88 ± 4.39	10.88 ± 0.89	19.88 ± 0.89
CT (n = 16)	16.81 ± 0.61	178.0 ± 3.5	61.89 ± 2.98	10.55 ± 1.01	19.53 ± 0.87

RT, resistance training; CT, complex training; BMI, body mass index (kg·m^-2^).

Throughout the study, participants maintained their regular training regimen, which included endurance training consisting of long-distance road running (70%–85% Maximal Heart rate (HRmax)) and interval training (90%–95% HRmax). Weekly training volume averaged 44.50 ± 6.33 km for endurance work, with total training time averaging 7.75 ± 0.97 h for endurance and 3.5 ± 1.5 h for strength training. The experimental protocols (complex training or resistance training) were integrated into the teams’ scheduled physical training sessions, conducted three times per week. Participants also continued their standard physical conditioning, including functional training, flexibility work, and injury prevention exercises. They were instructed to avoid additional strength training outside the study protocol. All strength sessions were supervised by certified strength and conditioning specialists. Post-testing was conducted after the 8-week intervention period, following the same procedures and conditions as the initial assessments.

### Measurements

All participants completed a 2-day testing protocol. On the first day they were tested for body composition, 1RM strength, CMJ, SJ and DJ, and on the second day they performed RE, VO_2max_ tests. The participants fasted for 2 hours before the test and wore the same running shoes during the pre- and post-tests.

#### Anthropometry and body composition test

Height was measured using a wall-mounted stadiometer (Butterfly, Shanghai, China) and recorded to the nearest 0.1 cm. Body mass, fat mass and fat-free mass were measured using a bioimpedance analyzer (X-scan plus II, Jawon, Daejeon, South Korea). Body mass index (BMI) was calculated as body mass divided by height squared.

#### Lower limb strength and power test

##### RM squat test

One RM testing that is properly administered has been found to be a valid and reliable measure of strength and power in children and adolescents (Faigenbaum et al., 2012). Lower-limb strength was assessed with a 1RM squat as reported by previous studies (Keiner et al., 2013). The maximal load of the parallel back-squat exercise (1RM) was determined using procedures outlined by National Strength and Conditioning Association (Miller, 2012). The parallel back squat exercise was performed following the same technique described for the squat training protocol. Before the 1RM assessment, participants completed a standardized warm-up consisting of four sets: (a) 10 repetitions at 20 kg, (b) five repetitions at 50% of the estimated 1RM, (c) three repetitions at 75% of the estimated 1RM, and (d) one repetition at 90% of the estimated 1RM. These progressive loads were designed to ensure adequate neuromuscular activation before maximal attempts. The estimated 1RM obtained during the preliminary recruitment session was used to determine the starting workload. Participants first attempted the estimated 100% 1RM, after which the load was increased by 5–10 kg until failure. Each participant was given up to six attempts to determine the actual 1RM. A standardized rest interval of 3 minutes between sets was provided to minimize fatigue and ensure reliable performance.

##### Vertical jump test

The vertical jump was used as a performance test to assess lower-limb power in adolescents, including the height of CMJ and SJ (Gavanda et al., 2022; Petrigna et al., 2019). After the 1RM test, each participant rested for 15 min, and then performed a SJ, CMJ and DJ test from a 40 cm height. Jumping height, peak power (maximum power during CMJ), foot contact time, and other related parameters were recorded using a force platform (Kistler 9281CA, KISTLER, Winterthur, Switzerland). To perform the CMJ, the participants were asked to stand on the force platform and place their hands on their hips. The participant then performed a rapidly downward squat movement and jumped vertically to attain maximum height. Arm-swing was not allowed during the jump. For the SJ, players were instructed to hold a static squat position with 90° knee flexion for 3 s before jumping. Three trials separated by 1 min of passive recovery were performed. The best trial for jump height was included in the data analysis.

Reactive strength, normally by measured by DJ height and reactive strength index (RSI) (Stricker et al., 2020), is defined as a runners’ capacity to efficiently utilize the stretch-shortening cycle (SSC) and elastic energy produced by the muscle–tendon complex (Beattie et al., 2017). For the DJ test, all participants were asked to stand on a 40 cm-high box and place their hands on their hips. The participants then stepped off the box to land on the force plate and jumped vertically for maximum height and minimum ground contact time. The trial was successful only when the participants did not bend the hip or knee during the jump and their hands did not leave the hips. Three trials separated by 1 min of passive recovery were performed. The best trial for jump height was included in the data analysis. The RSI was calculated by dividing jumping height in cm by contact time in seconds.

Pre-stretch augmentation percentage (PSAP) was used to indirectly examine the ability of an athlete to use the SSC to improve their jump height and peak power during a vertical jump, which was often used as an indicator of lower-limb power performance (Stricker et al., 2020). Indices from the jump data were PSAP and were calculated as follows:

$$\text{PSAP}=\frac{\text{CMJheight}-\text{SJheight}}{\text{SJheight}}\times 100\%$$

#### Running economy and related physiological tests

All physiological variables (VO_2max_, RE, and blood lactate concentration [BLa]) were measured using the treadmill protocol (Li et al., 2019) (Life Fitness T5, Rosemont, Illinois, United States). For use with adolescents, this type of test has proven effective (Mikkola et al., 2007). Oxygen uptake and heart rate (HR) were determined using a portable metabolic analyzer (K5, Cosmed Srl, Rome, Italy) and HR monitor belt (Garmin, Olathe, Kansas, United States). Finger capillary blood was collected prior to the test to confirm that each participant was in a normal physiological state. Blood samples were analyzed for lactate concentration, and only participants with resting blood lactate levels below 2 mmol L^-1^ were considered to be in a normal, non-fatigued condition. The subject then warmed-up on the treadmill set to 8 km h^−1^ for 10 min. After the warm-up period, the subject rested for 5 min and then began a 4-min run at each of three incremental submaximal speeds (12, 14, and 16 km h^-1^, respectively), which were below the maximum metabolic steady-state (corresponding to a blood lactate concentration ≤4 mmol L^-1^ or below the critical speed threshold) intensity to ensure that running economy primarily reflected aerobic energy expenditure. This RE testing protocol was similar to that in previous studies (Sedano et al., 2013) and reflected the runners’ ability to run at submaximal speeds. Participants ran for 4 min to ensure adequate time for their VO_2_, HR, and BLa to reach a steady state (Beattie et al., 2017; Saunders et al., 2006). After each 4-min stage of running, the subject rested for 1 min, during which finger blood samples were collected. All finger blood samples were used to measure their blood lactate concentrations and aerobic capacity during running via a lactate analyzer (EKF Diagnostic, Magdeburg, Germany).

After the completion of the last stage of the running economy test, the treadmill speed was set to 17 km h^−1^, which was then increased by 1 km h^−1^every 2 min, until the subject reached exhaustion. The following criteria were used to determine exhaustion: heart rate greater than 90% of age-predicted maximal HR (calculated by 220 –runner’s age); respiratory exchange ratio (RER) ≥1.10; and rating of perceived exertion (RPE) above 18. VO_2max_ was determined as the highest VO_2_ value using a 30s moving window.

### Training program

Before beginning the main training intervention, all adolescent runners completed a 2-week preparatory phase (two sessions per week) focused on developing fundamental movement patterns, including squat, hip-hinge, and upper-body push/pull exercises, to ensure safe execution of free-weight resistance training.

The subsequent 8-week training intervention consisted of three supervised sessions per week (60 min each) with at least 48 h of recovery between sessions. Tables 2, 3 present the detailed structure and progression of the CT and RT programs.

**TABLE 2:** Complex training program.

Exercises	Stage 1 (Week 1–2)	Stage 2 (Week 3–5)	Stage 3 (Week 6–8)
Complex pair 1	Back Squat (60%–65%1RM × 6-12RM × 3sets) Box Jump (9–12 reps/set × 3 sets)	Back Squat (70%–75%1RM × 6-12 RM × 3 sets) Vertical Jump (9–12 reps/set × 3 sets)	Back Squat (80%1RM × 8-12RM × 4sets) Drop Jump (10–12 reps/set × 4 sets)
Complex pair 2	Hexagonal Barbell Pull-up (60%–65%1RM × 6-12RM × 3 sets) Double-legs Hurdle Hop(30 cm) (9–12 reps/set × 3sets)	Hexagonal Barbell Pull-up (70%–75%1RM × 6-12RM × 3 sets) Double-legs Hurdle Hop(35 cm) (9–12 reps/set × 3 sets)	Hexagonal Barbell Pull-up (80%1RM × 6-12RM × 4 sets) Double-legs Hurdle Hop(40 cm) (10–12reps/set × 4sets)
Complex pair 3	Weight-bearing Bulgarian Squat (60%–65%1RM × 6-12RM × 3 sets) Split-leg Squat Jump (9–12reps/leg/set × 3 sets)	Weight-bearing Bulgarian Squat (70%–75%1RM × 6-12RM × 3 sets) Single-legged Side Box Jumps (9–12 reps/leg/set × 3 sets)	Weight-bearing Bulgarian Squat (80%1RM × 6-12RM/leg × 4 sets) Single-legged Drop Jump (10–12 reps/leg/set × 4 sets)
Complex pair 4	Weight-bearing Heel-lifting (60%–65%1RM × 6-12RM × 3 sets) Jump on Tiptoe (9–12 reps/set × 3 sets)	Weight-bearing Heel-lifting (70%–75%1RM × 6-12RM × 3 sets) Jump on Tiptoe (9–12 reps/set × 3 sets)	Weight-bearing Heel-lifting (80%1RM × 6-12RM × 4 sets) Jump on Tiptoe (10–12 reps/set × 4 sets)
Rest	Pairs:4 min rest interval Between groups: 3–4 min rest	Pairs:4 min rest interval Between groups: 3–4 min rest	Pairs:4 min rest interval Between groups: 3–4 min rest

RM, maximum repetitions; % 1RM, percentage of 1RM, maximum load intensity.

**TABLE 3:** Resistance training program.

Exercises	Stage 1 (Week 1–2)	Stage 2 (Week 3–5)	Stage 3 (Week 6–8)
Complex pair 1	Back Squat (60%–65% 1RM × 6–12 RM × 3 sets; 4 sets for RT group)	Back Squat (70%–75% 1RM × 6–12 RM × 3 sets; 4 sets for RT group)	Back Squat (80% 1RM × 8–12 RM × 4 sets; 5 sets for RT group)
Complex pair 2	Hexagonal Barbell Pull-up (60%–65% 1RM × 6–12 RM × 3 sets; 4 sets for RT group)	Hexagonal Barbell Pull-up (70%–75% 1RM × 6–12 RM × 3 sets; 4 sets for RT group)	Hexagonal Barbell Pull-up (80% 1RM × 6–12 RM × 4 sets; 5 sets for RT group)
Complex pair 3	Weight-bearing Bulgarian Squat (60%–65% 1RM × 6–12 RM × 3 sets/leg; 4 sets for RT group)	Weight-bearing Bulgarian Squat (70%–75% 1RM × 6–12 RM × 3 sets/leg; 4 sets for RT group)	Weight-bearing Bulgarian Squat (80% 1RM × 6–12 RM × 4 sets/leg; 5 sets for RT group)
Complex pair 4	Weight-bearing Heel-lifting (60%–65% 1RM × 6–12 RM × 3 sets; 4 sets for RT group)	Weight-bearing Heel-lifting (70%–75% 1RM × 6–12 RM × 3 sets; 4 sets for RT group)	Weight-bearing Heel-lifting (80% 1RM × 6–12 RM × 4 sets; 5 sets for RT group)
Rest	Rest between resistance and plyometric exercises within each complex pair: 3–4 min	Rest between resistance and plyometric exercises within each complex pair: 3–4 min	Rest between resistance and plyometric exercises within each complex pair: 3–4 min

RM, maximum repetitions; % 1RM, percentage of 1RM, maximum load intensity.

Participants in the CT group performed paired resistance and plyometric exercises targeting similar movement patterns. The resistance training volume (2–4 sets × 6–12 reps, <80% 1RM) followed the International Consensus on Youth Resistance Training (Lloyd et al., 2014) and other studies (Stricker et al., 2020). Progression was based on successful completion of two or more additional repetitions in the final set of two consecutive sessions, after which the load was increased to maintain the target repetition range (8–12 reps) (McKinlay et al., 2018). Plyometric training volume (3–4 sets × 9–12 foot contacts per exercise) and intensity (box height, movement complexity) were progressed according to technical competency and amortization phase duration, in line with prior recommendations (Lesinski et al., 2016; Faigenbaum and Myer, 2010). The intra-complex rest interval was 4 min, with 3–4 min between sets.

Each training session began with a standardized 15-min dynamic warm-up, followed by lower-limb exercises such as squats, 45° leg presses, and hip thrusts. The RT group performed the same resistance exercises as the CT group but excluded plyometric work; to equate total volume, one additional set was added to the final exercise. Resistance exercises were performed at controlled velocities (2-s eccentric, 1-s pause, 2-s concentric), while plyometric exercises were executed as explosively as possible.

### Statistical analysis

Statistical analysis was performed using IBM SPSS software (version 26.0, Chicago, IL, United States). Data are presented as mean ± standard deviation (M ± SD). The Shapiro–Wilk test was applied to verify normality, and outliers (studentized residuals >3 SD from zero) were removed. The effects of complex training on strength and performance parameters were analyzed using two-way repeated-measures ANOVA (group × time), with dependent variables including VO_2_max, RE (12, 14, and 16 km h^-1^), BLa (12, 14, and 16 km h^-1^), HR (12, 14, and 16 km h^-1^), 1RM squat, CMJ, CMJ peak power, SJ, PSAP, DJ, and RSI. When significant interactions were detected, LSD *post hoc* tests were conducted. Within-group effects were examined using one-way ANOVA with time as the factor. Effect sizes (partial η^2^) were interpreted as small (<0.06), moderate (<0.14), or large (≥0.14) (Cohen, 1988).

## Results

**FIGURE 2:**
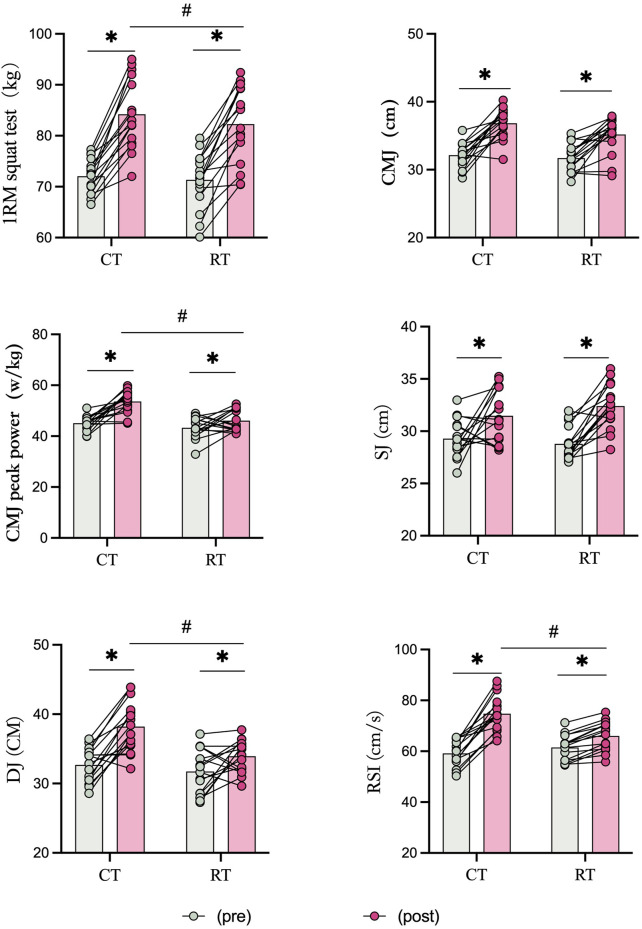
The running economy task performance before and after Complex Training (CT) and Resistance Training (RT). * Statistically significant difference between pre-and post-test, p < 0.05.# Statistically significant difference between group, p < 0.05.

**FIGURE 3:**
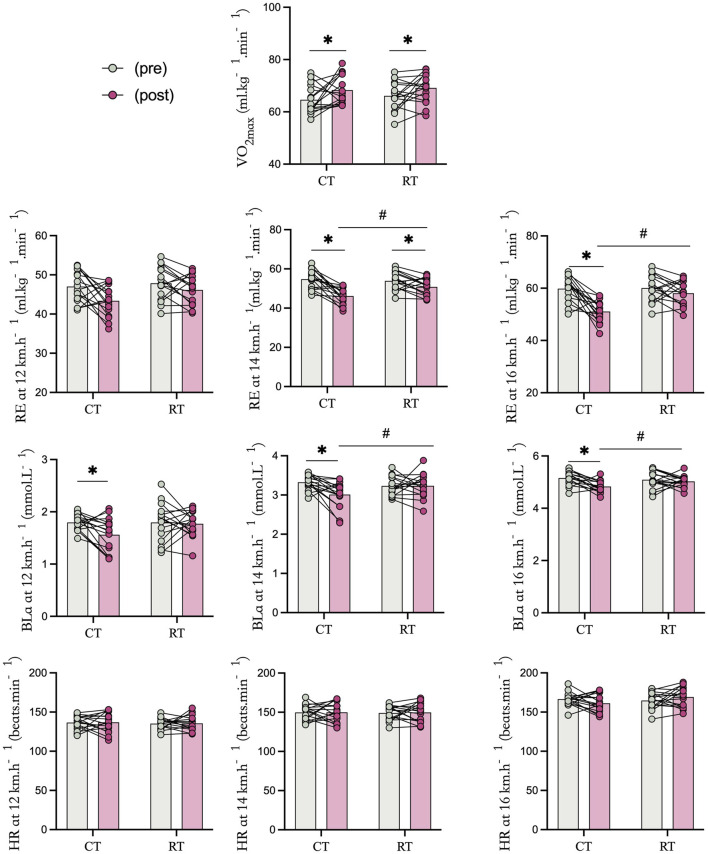
The strength and power task performance before and after Complex Training (CT) and Resistance Training (RT). * Statistically significant difference between pre-and post-test, p < 0.05.# Statistically significant difference between group, p < 0.05.

All participants (N = 32) completed the study, and their data were included in the analysis. Data were normally distributed, with no significant baseline differences between groups in demographic characteristics, strength, power, or performance measures (p > 0.05). The main results, including ANOVA outcomes, descriptive statistics, and effect sizes, are presented in Table 4, while individual and mean values before and after the interventions are illustrated in Figures 2, 3.

**TABLE 4:** Results of CT and traditional RT before and after 8 weeks of training.

Ability	Indicator	CT group				RT group			
		Pre	Post	*p*	Partial $\eta^{2}$	Pre	Post	*p*	Partial $\eta^{2}$
Running economy	VO_2max_ (mL.kg^−1^.min^−1^)	64.92 ± 5.18	68.34 ± 5.65*	<0.001	0.643	65.84 ± 5.95	69.17 ± 5.56*	<0.001	0.631
	RE at 12 km h^−1^ (mL.kg^−1^.min^−1^)	47.05 ± 4.04	43.38 ± 3.972*	<0.001	0.761	47.81 ± 4.32	46.15 ± 3.78*	<0.001	0.395
	RE at 14 km h^−1^ (mL.kg^−1^.min^−1^)	54.99 ± 4.69	46.15 ± 4.23*#	<0.001	0.547	59.76 ± 5.40	58.07 ± 4.74*	<0.001	0.133
	RE at 16 km h^−1^ (mL.kg^−1^.min^−1^)	59.81 ± 4.89	51.07 ± 4.20*#	<0.001	0.516	60.25 ± 5.06	58.07 ± 4.73	0.210	0.052
	BLa at 12 km h^−1^ (mmol.L^−1^)	1.77 ± 0.11	1.48 ± 0.25*	0.014	0.184	1.80 ± 0.36	1.79 ± 0.15	0.774	0.003
	BLa at 14 km h^−1^ (mmol.L^−1^)	3.32 ± 0.18	3.01 ± 0.34*	0.002	0.287	3.23 ± 0.23	3.23 ± 0.30	0.973	0.000
	BLa at 16 km h^−1^ (mmol.L^−1^)	5.16 ± 0.0.26	4.83 ± 0.22*#	0.001	0.292	5.09 ± 0.34	5.02 ± 0.22	0.486	0.016
	HR at 12 km h^−1^ (beats.min^−1^)	136.62 ± 8.99	136.94 ± 12.60	0.632	0.632	135.31 ± 7.16	135.63 ± 9.55	0.969	0.003
	HR at 14 km h^−1^ (beats.min^−1^)	149.75 ± 9.86	149.94 ± 12.86	0.960	0.000	149.12 ± 9.36	149.81 ± 12.39	0.924	0.000
	HR at 16 km h^−1^ (beats.min^−1^)	167.94 ± 8.60	161.31 ± 11.72	0.141	0.071	164.75 ± 10.59	169.318 ± 12.87	0.217	0.150
Strength and power	1RM squat test (kg)	72.04 ± 3.39	84.20 ± 6.84*	<0.001	0.724	71.36 ± 5.45	82.28 ± 7.46*	<0.001	0.679
	CMJ (cm)	32.13 ± 1.81	36.84 ± 2.16*	<0.001	0.634	31.72 ± 2.02	35.18 ± 2.65*	<0.001	0.482
	CMJ peak power (w/kg)	45.13 ± 2.9	53.63 ± 4.40*#	<0.001	0.534	43.27 ± 4.16	46.10 ± 4.23*	0.060	0.113
	SJ (cm)	29.29 ± 1.77	31.47 ± 2.51*	0.005	0.232	28.79 ± 1.57	32.40 ± 2.11*	<0.001	0.453
	DJ (cm)	32.70 ± 2.43	38.20 ± 3.56*#	<0.001	0.542	31.75 ± 3.18	33.94 ± 2.32*	0.024	0.159
	RSI(cm/s)	59.18 ± 5.02	74.79 ± 6.93*#	<0.001	0.842	61.48 ± 4.99	66.06 ± 5.65*	0.001	0.314

Abbreviations: VO_2_max, maximal oxygen uptake; RE, running economy; BLa, blood lactate concentration; HR, heart rate; 1RM, one-repetition maximum; CMJ, countermovement jump; SJ, squat jump; DJ, drop jump; RSI, reactive strength index.

p *< 0.05*, significant difference between pre- and post-test; *#p < 0.05*, significant difference between groups.

### Running economy

Two-way repeated-measures ANOVA revealed significant group × time interactions for running economy at all speeds (12 km h^-1^: *p* = 0.001; 14 km h^-1^: *p* = 0.010; 16 km h^-1^: *p* = 0.004) and for blood lactate at 14 km h^-1^ (*p* = 0.019). However, no significant interactions were found for VO_2_max (*p* = 0.894), blood lactate at 12 km h^-1^ (*p* = 0.113) and 16 km h^-1^ (*p* = 0.056), or heart rate at any speed (*p* > 0.05).

Post-hoc analyses showed that the CT group achieved significantly greater improvements than the RT group in running economy at all three speeds (12 km h^-1^: *F* = 19.580, *p* < 0.001, η^2^ = 0.395, large effect; 14 km h^-1^: *F* = 36.205, *p* < 0.001, η^2^ = 0.547, large effect; 16 km h^-1^: *F* = 31.937, *p* < 0.001, η^2^ = 0.516, large effect) and in blood lactate at 14 km h^-1^ (*F* = 12.077, *p* = 0.002, η^2^ = 0.308, moderate effect). Both groups exhibited significant improvements in VO_2_max (CT: *p* < 0.001; RT: *p* < 0.001, large effects). For blood lactate, the CT group showed significant reductions at 12 km h^-1^ (*p* = 0.014, moderate effect) and 14 km h^-1^ (*p* = 0.001, large effect), whereas the RT group showed no significant changes (12 km h^-1^: *p* = 0.774; 16 km h^-1^: *p* = 0.486, small effects).

### Strength and power

For strength and power measures, significant group × time interactions were observed for CMJ peak power (p = 0.010, moderate effect), drop jump (DJ) performance (p = 0.017, moderate effect), and reactive strength index (RSI; p < 0.001, large effect), while no significant interactions were found for 1RM squat (p = 0.525), CMJ height (p = 0.185), or squat jump (SJ; p = 0.173).

Post-hoc analyses revealed significantly greater improvements in the CT group compared with the RT group for CMJ peak power (F = 66.627, p < 0.001, η^2^ = 0.690, large effect), DJ performance (F = 35.542, p < 0.001, η^2^ = 0.542, large effect), and RSI (F = 39.862, p < 0.001, η^2^ = 0.571, large effect). Both groups demonstrated significant improvements in 1RM squat test, CMJ height, and SJ performance (all p < 0.001, large effects).

## Discussion

This study demonstrated that CT can effectively improve both running economy and lower-limb strength in adolescent distance runners, offering advantages over traditional RT. These findings provide important insights into optimizing strength and conditioning strategies for this specific developmental stage.

The complex training group showed significant improvements in running economy at all tested speeds (12, 14, and 16 km/h), particularly at higher velocities. These results are consistent with findings from adolescent and adult endurance runners, where resistance or combined resistance–plyometric training improved running economy through enhanced neuromuscular efficiency and mechanical stiffness (Beattie et al., 2017; Blagrove et al., 2018; Barnes and Kilding, 2015). Compared with traditional resistance training, complex training—which combines heavy resistance and plyometric exercises—may induce superior neuromuscular and mechanical adaptations. Traditional resistance training primarily enhances maximal strength and muscle-tendon stiffness, which can improve running economy by reducing energy cost during submaximal exercise (Beattie et al., 2017). In contrast, the addition of plyometric exercises in complex training further stimulates neural adaptations, including improved motor unit synchronization, increased rate of force development, and reduced antagonist co-activation (Markovic and Mikulic, 2010). These adaptations enhance the efficiency of the stretch–shortening cycle and facilitate greater elastic energy reutilization, leading to more economical force production and improved running efficiency. Consequently, complex training may provide greater improvements in running economy and endurance performance compared with traditional resistance training alone.

The observed reduction in blood lactate concentration at submaximal speeds further supports improved metabolic efficiency. Similar adaptations have been reported in trained adult runners following strength-based interventions, where reduced lactate accumulation indicated lower reliance on anaerobic metabolism at a given running intensity (Barnes and Kilding, 2015; Fletcher and MacIntosh, 2017). For adolescent runners, this shift suggests enhanced oxidative capacity and delayed fatigue onset, both of which are critical for endurance performance development during growth.

Both training protocols significantly increased maximal strength (1RM squat) and jump performance (CMJ and SJ), confirming that structured resistance training is effective for improving basic strength parameters in adolescents (Lloyd et al., 2016; Faigenbaum et al., 2016). However, the complex training group achieved greater improvements in power-related measures, particularly CMJ peak power, DJ height, and RSI. These results align with studies in both youth and adult endurance runners, indicating that combining heavy-load resistance and plyometric exercises enhances muscle-tendon stiffness and rate of force development more effectively than resistance training alone (Maio Alves et al., 2010; Beattie et al., 2017).

The improvement in RSI reflects enhanced utilization of elastic energy and faster transition between eccentric and concentric muscle actions—key factors contributing to running economy, particularly at higher speeds where the efficiency of the stretch-shortening cycle becomes increasingly decisive (Fletcher and MacIntosh, 2017). This mechanistic link between neuromuscular performance and running economy provides empirical support for the integration of complex training in adolescent endurance training programs.

Although significant improvements were observed in strength and running economy, no meaningful change occurred in VO_2_max. This outcome is consistent with previous findings in adolescent and adult endurance athletes, where resistance-based interventions primarily enhanced running economy and strength rather than maximal oxygen uptake (Denadai et al., 2017; Beattie et al., 2017). The absence of a VO_2_max increase may be attributed to the short intervention duration (8 weeks), the non-endurance nature of the training stimulus, and the specificity principle—strength and plyometric training primarily target neuromuscular efficiency rather than central cardiorespiratory adaptations. Thus, the improvement in running economy without VO_2_max change suggests that submaximal performance benefits are largely mediated by peripheral neuromuscular and biomechanical adaptations rather than central aerobic capacity improvements.

The findings support incorporating CT into adolescent runners’ programs, emphasizing gradual progression, technical mastery, and adequate recovery between sessions (Lloyd et al., 2014; Lesinski et al., 2016). The CT appears particularly suitable during preparatory phases when building fundamental physical qualities is prioritized. Coaches should systematically monitor both strength and endurance parameters to individualize training loads and optimize adaptation.

## Limitations

Several limitations should be acknowledged. First, the relatively short training duration (8 weeks) may not fully reflect long-term adaptations or retention effects. Second, the exclusive inclusion of male adolescent runners limits the generalizability of findings to female athletes or other age categories. Future studies should explore optimal periodization strategies, investigate sex-based differences in adaptation, and examine long-term effects of complex training on endurance performance, biomechanics, and injury prevention. Furthermore, advanced neuromuscular assessments (e.g., muscle stiffness, EMG activation patterns) and biomechanical analyses could help clarify the specific mechanisms underlying improvements in running economy.

## Conclusion

This study provides evidence that complex training is superior to traditional resistance training for improving both running economy and power-related performance measures in adolescent distance runners. The findings suggest that the integration of heavy resistance exercises with plyometric movements creates synergistic adaptations that enhance both strength and running efficiency. These results have important implications for coaches and practitioners working with adolescent distance runners, providing support for the inclusion of complex training in their preparation programs.

## Data Availability

The original contributions presented in the study are included in the article/supplementary material, further inquiries can be directed to the corresponding authors.
